# Viral infections and wheezing–asthma inception in childhood: is there a role for immunomodulation by oral bacterial lysates?

**DOI:** 10.1186/s13601-020-00322-1

**Published:** 2020-06-03

**Authors:** Giovanni A. Rossi, Petr Pohunek, Wojciech Feleszko, Stefania Ballarini, Andrew A. Colin

**Affiliations:** 1 Department of Pediatrics, Pulmonary and Allergy Disease Unit and Cystic Fibrosis Center, G. Gaslini University Hospital, Largo G. Gaslini, 4, 16148 Genoa, Italy; 2 Dept of Paediatrics, 2nd Faculty of Medicine, Charles University, Prague, Czech Republic; 3 Department of Pediatric Pulmonology and Allergy, The Medical University of Warsaw, Warsaw, Poland; 4 Medical Affairs Lead, Infectious Diseases, OM Pharma, a Vifor Pharma Company, Meyrin, Geneva, Switzerland; 5 Division of Pediatric Pulmonology, Miller School of Medicine, University of Miami, Miami, FL USA

**Keywords:** Bacterial lysate, Immune system, Allergy

## Abstract

Severe and recurrent infections of the respiratory tract in early childhood constitute major risk factors for the development of bronchial hyper-responsiveness and obstructive respiratory diseases in later life. In the first years of life, the vast majority of respiratory tract infections (RTI) leading to wheezing and asthma are of a viral origin and severity and recurrence are the consequence of a greater exposure to infectious agents in a period when the immune system is still relatively immature. Therefore, boosting the efficiency of the host immune response against viral infections seems to be a rational preventative approach. In the last decades it has been demonstrated that living in farm environments, i.e. early-life exposure to microbes, may reduce the risk of allergic and infectious disorders, increasing the immune response efficacy. These findings have suggested that treatment with bacterial lysates could promote a nonspecific immunomodulation useful in the prevention of recurrent RTIs and of wheezing inception and persistence. Experimental and clinical studies showing the reduction of RTI frequency and severity in childhood and elucidating the involved mechanisms can support this hypothesis.

## Background

Respiratory tract infections (RTI) are amongst the leading causes of childhood morbidity and mortality globally [1, 2]. Viruses, and less often bacteria, are involved in the pathogenesis and frequently the etiology is mixed [3, 4]. Most children with recurrent RTI are otherwise healthy and the respiratory recurrences are the consequence of increased early life exposure to infectious agents, prior to full maturation of the immune system [5–7]. Most acute RTI are self-limiting, but often recur and are associated with respiratory sequelae, most commonly airway hyper-responsiveness, wheezing and ultimately asthma [7–9]. International guidelines for prevention or treatment of these highly prevalent clinical problems are scanty [10]. Over the last decades, attempts have been made to reduce post-infectious respiratory symptoms and morbidity by using inhaled corticosteroids or leukotriene receptor antagonists, but no disease-modifying effects have been documented [11, 12]. It was, however, observed that living in farming communities may increase the efficiency of the immune responses, not only against allergens but possibly also against infectious pathogens [13]. The evidence that early-life exposure to microbes may have protective effects has suggested that prebiotics, probiotics, and bacterial lysates could prevent the onset of wheezing and asthma through “nonspecific immunomodulation” of the body’s natural defenses [14] and reduce virus-induced wheezing exacerbations [15]. It has been even hypothesized that oral bacterial lysates (OBL) may promote a re-organization not only of the gut but also of the lung microbiota thus favouring the immune homeostasis via the gut-lung axis [16] This is an area of future research, identified also by National Heart, Lung, and Blood Institute roadmap, to provide potential targets of intervention in the primary prevention of chronic lung diseases [17]. In the present review, we will summarize and discuss the available data on how pharmaceutical products containing bacterial antigens can modulate the host immune response in children and could represent a valuable option in the prevention of recurrent RTI, in instances attributable to a viral origin. Early evidence suggests that bacterial lysates might play a role in reducing the risk of recurrent wheeze and possibly of asthma inception filling a gap in this area of unmet high medical need.

## Viral infections, wheezing and asthma

Structural and functional changes induced by respiratory viral infections in the airways during early childhood may promote self-perpetuating inflammatory mechanisms and, in predisposed individuals, a selection towards a Th2-type response that can lead to wheezing and asthma. Highly sensitive and specific tests, such as polymerase chain reaction, have been developed in the past few decades, allowing accurate detection of viruses associated with acute and recurrent wheezing illness [15, 18, 19]. This has led to a better understanding of the pathogenesis of these disorders as well as the features of the innate and adaptive immune response, here summarized, that can be modulated by therapeutics such as bacterial lysates. Respiratory syncytial virus (RSV) and human rhinovirus (HRV) top the list of viruses involved in the pathogenesis, but enterovirus, human metapneumovirus, bocavirus and coronavirus also play a role [15, 18–22]. In particular, the recent SARS-CoV-2 coronavirus pandemic made us aware of possible serious public health consequences associated with infection with a respiratory virus apparently relatively benign in children but possibly devastating when being spread out to adults [23]. All these respiratory viruses target airway epithelial cells [3, 15, 20]. Viral adhesion to the epithelial cell membrane is followed by infection, viral replication, virion shedding/budding into the airway lumen and infection of neighboring cells [19, 20]. Recognition of viral transcripts and replication intermediates by host cells induces production of cytokines and chemokines, aimed at mounting an effective early antiviral response. In the first days of the infection, recruited natural killer (NK) cells and polymorphonuclear leukocytes eliminate the infected epithelial cells, limiting viral replication and spread [20, 23]. The resulting epithelial damage is associated with bronchial inflammation and hyperresponsiveness [24]. Respiratory viruses also infect and/or are recognized by airway dendritic cells (DCs) that migrate to regional lymphoid tissues and present viral antigens to naïve T-cells [20, 21, 25–27]. Depending on the immunological environment, naïve T-cells differentiate into Th1 or Th2 cells. Th1 cell polarization promotes interferon-(IFN)-γ production with activation of CD8+ cytotoxic T-cells and NK cells to clear viruses and the infected host cells by direct cytolysis. In contrast, Th2 cell polarization results in an inefficient immune response, increased disease severity and risk for future recurrent wheezing [25]. Upon contact with antigen-presenting cells, macrophage activities are also stimulated, and B-lymphocyte proliferation and differentiation induced, with production of antigen-specific IgM, IgG, and IgA. Two other T-lymphocyte populations, derived from naïve CD4+ T-cells, are involved in the response to viral infection: regulatory T-cells (Treg) and interleukin IL-17-producing T helper cells (Th17). Treg cells are involved in promotion of viral clearance as well as modulation of many aspects of the innate and adaptive immune responses. These include activation of neutrophils and NK cells, early recruitment of CD8+ cytotoxic T-cells, but also prevention of an excessive antigen-specific CD4+ and CD8+ T-cell response and, importantly, limitation of inefficient Th2-type immune reaction, through the production of the inhibitory cytokines IL-10 and transforming growth factor-(TGF)-β [27–29]. Th17 cells modulate Treg cell activities and increase host resistance to virus and bacteria, playing a positive role in the preservation of mucosal barrier integrity and, at later time points, in promoting recovery of normal architecture and lung function [30]. Indeed, IL-17 and IL-22, cytokines secreted by Th17 cells, are not only critical for host defense at mucosal surfaces, but also for tissue repair. An in-depth analysis of the complex interactions that occur in viral infections between Treg and Th17 cells is beyond the scope of this review. Infected airway epithelial cells may also promote a Th2 polarization, with recruitment-activation of mast cells, eosinophils and basophils, cells that have no identified role in clearing viral infection [20, 21, 25]. On airway epithelial cells, Th2 cytokines increase expression of adhesion molecules for HRVs, the predominant viruses producing recurrent wheezing and asthma exacerbation in children [31]. These include intercellular adhesion molecule (ICAM)-1, which acts as receptor for both type A and B HRVs, and cadherin-related family member 3 (CDHR3), which mediates type C HRVs binding and replication [19–22, 31–33]. In conclusion, independently from allergen exposure, some viruses, particularly type A and C HRV, may induce severe damage to the airways and a type 2 immune responses. The acquired Th2 phenotype polarization can subsequently become permanently skewed, if sustained by recurrent infections, and favored by environmental factors and by changes in microbiota composition [19, 27, 34, 35].

## Physiological immaturity of the immune response

**Fig. 1:**
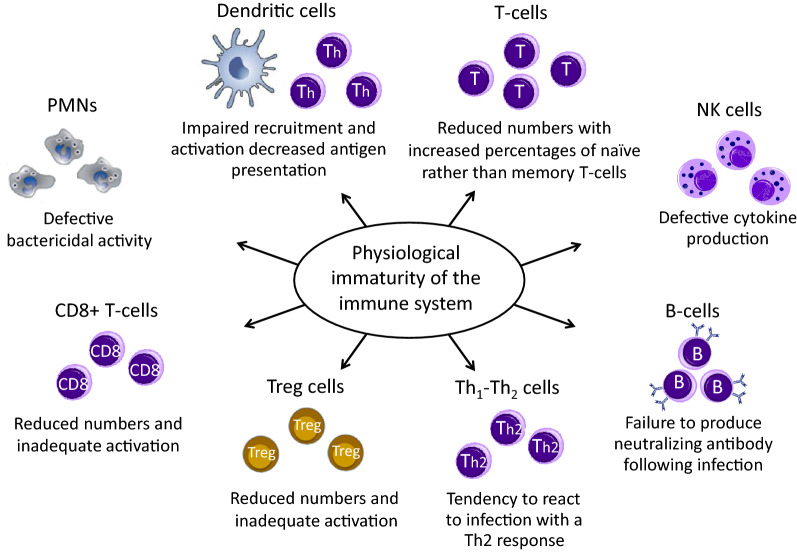
Negative effects of immaturity of the immune cells in young children less than 3 years old. Both the innate and the adaptive arms of the immune system go through extended periods of post-natal maturation that persist over the first years of life, increasing the susceptibility to recurrent and severe viral respiratory infections

The increased susceptibility of infants and young children to respiratory infections is the result of the physiological immaturity of components of the systemic and local immune responses and, possibly, of sub-optimal complex inter-talk between microbiota and immune system effectors [4, 13, 14]. Immunotherapies that can modulate the immune response acting on immune cells and pathways as well as on gut microbiota are therefore desirable in childhood [36]. Both innate and adaptive arms of the immune system go through extended periods of post-natal maturation (Fig. 1). Neutrophils from neonates exhibit defective bactericidal activity [36–39]. NK cell proportions are not reduced in cord blood, but their IFN-γ production and cytotoxic activity are weaker [25, 26, 40]. The lower DC efficiency contributes to sustain the early “at birth” Th2 bias, related to elevated intrauterine IL-4 and IL-10 production [36]. The TH1/Th2 imbalance tends to persist over the first years of life, increasing the susceptibility to severe viral infections [36–39]. Reduced mobilization of DC precursors from bone marrow has also been described in neonates, associated with weak activation of the type I IFN pathways, low secretion of cytokines and decreased ability to present antigens to T-cells. These deficiencies are, at least in part, related to the reduced expression of surface proteins, involved in antigen presentation: the major histocompatibility complex II (MHC II) molecules and the CD40 and CD86 co-stimulatory molecules [27, 35, 39, 41, 42]. Naïve T-cells have non-specific receptors, responding to a wide range of peptides, as opposed to the highly-specific-antigen receptors of mature T-cells [27, 35, 39, 41, 42]. This explains the defective production of antigen-specific neutralizing antibodies by B-lymphocytes in response to infections [43]. Also, antigen-presenting B-lymphocytes lack MHC II and co-stimulatory CD40 and CD86 signals, leading to inefficient interactions with T-cells and antibody isotype switch [44]. The low titers of protective antibody, characterizing infancy, persist reaching adult levels only around adolescence [44, 45]. In young children Treg cells are inefficient as well, since they lack functional signals needed to promote viral clearance and prevent allergic sensitization [46]. Treg cells have been shown to play a critical role in preventing disease in RSV-infected mice, inhibiting lung inflammation and immune-mediated pathology by modulating CD8 T-cell response [47]. In RSV-infected infants, a reduction in abundance of activated Treg cells has been reported that persisted as the infection progressed and was associated with elevated levels of inflammatory cytokines in the nasal aspirates, increased neutrophilic inflammation, and exaggerated mucus production [48]. Allergen-specific Treg cells are also involved in prevention of atopic sensitization, through induction of a state of immune tolerance to inhaled allergens [49]. A key role in the maturation and modulation of the immune system is also thought to be played by the gut microbiota, where low diversity and/or dysbiosis in this phase of life can affect respiratory health [13, 14, 50].

## Gut microbiota

Early-life gut microbiota development has proven to be a critical factor in protecting against infections [50–52]. Recent evidence shows that gut commensal non-pathogenic species may influence immune response in other organs, notably airways [50–52]. This may be achieved through competitive colonization, direct effects of microbiota metabolic products on somatic functions, or through the activation of protective immune mechanisms. The gut microbiota develops immediately after birth, increases dramatically in diversity in the first three years of life and stabilizes thereafter to the adult pattern [51, 52]. Therefore, infancy represents a critical period in which the gut microbiota can be positively or negatively modified, with obvious implications for health outcomes. It has been hypothesized that inadequate maturation of the gut microbiome in early childhood, causing immune dysregulations, could potentially promote asthma in predisposed children [53, 54]. Amongst the variety of events that can negatively affect microbiota, a primary role is played by exposure to antibiotics [55–58]. Antibiotic use is associated with decreased total bacterial density, increased detection of gram-negative pathogenic bacilli and reduction in genera associated with fewer respiratory illnesses [55]. In addition to having a detrimental impact on the microbiota, inappropriate prescription of antibiotic treatments for viral infections can increase drug resistance among pathogenic bacteria [58]. Administration of bacterial extracts was shown to reduce antibiotic use by lowering risk of infection [14]. Furthermore, because of their nature, when given orally, they might also play a direct beneficial role on the gut microbiota thus also promoting respiratory health during the first years of life.

## Oral bacterial lysates

**Fig. 2:**
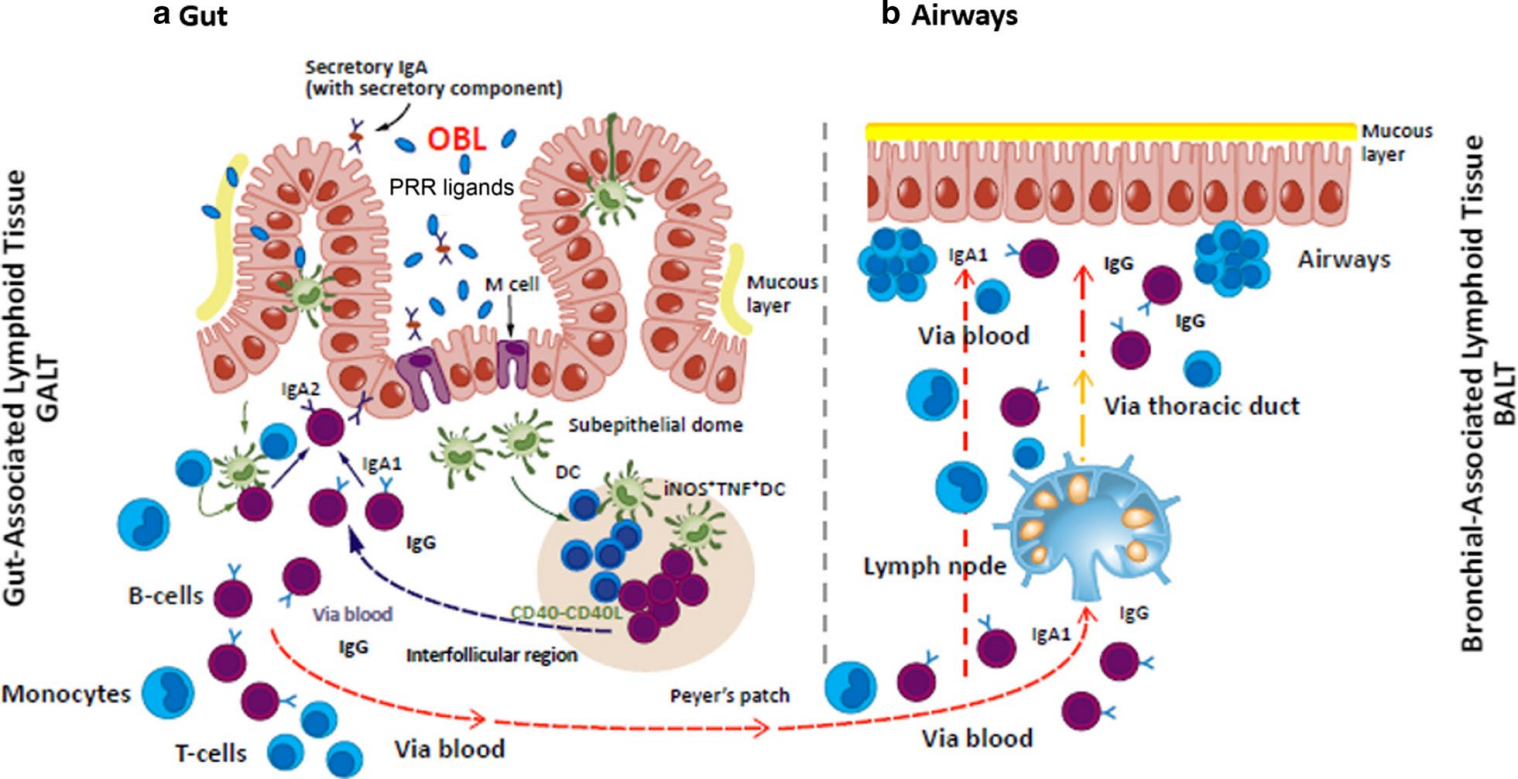
After oral administration of bacterial lysates (OBL), bacterial antigens are taken up by microfold (M) cells and then processed by dendritic cells (DC) in the gut-associated lymphoid tissue. DC stimulate the local immune response in the Peyer’s patches with subsequent migration of immune effector cells to the bronchial-associated lymphoid tissue of the lungs. From ref. #[61], modified with permission

Several bacterial lysates available for clinical use have shown to provide clinical benefit in patients with recurrent RTIs [14]. We will report and discuss only results of pre-clinical and clinical studies related to the “orally-delivered” bacterial lysates, whose mechanisms of action involve stimulation of the gut-associated lymphoid tissue and potentially promoting cross-talks between gut microbiota and immune system effectors. We will not include in this review Pidotimod, an orally delivered synthetic thymic dipeptide, and Polyvalent Mechanical Bacterial Lysate that is delivered by sublingual route [14]. The three key features of OBL supporting their use in prevention of viral infections and wheezing–asthma exacerbations are: (a) activation of a broad non-specific anti-infectious innate immune response; (b) prevention of excessive inflammatory response; (c) promotion of an adaptive Th1 type immune response with inhibition of an inefficient Th2-type polarization [14]. OBL are mechanical or chemical extracts of different strains of inactivated pathogenic respiratory bacteria species involved in the etiology of RTI. They contain conserved bacterial “pathogen-associated molecular patterns” (PAMPs), recognized by cellular “pattern recognition receptors” (PRRs), expressed by cells of the innate and adaptive immune responses [14, 59, 60]. Extracts that contain lysates from multiple bacterial strains, derived from different bacterial species, use bacterial diversity, as well as commonality preserved across the evolution of microorganisms, to act as PRR ligands. These active components expose the immune system to triggers like those that are carried by live infectious and commensal microorganisms, mimicking the natural exposure occurring in life, with consequent generation of a polyvalent immunomodulation [14, 60]. PRR-mediated pathogen recognition activates transcriptional pathways that promote the production of molecules required for a rapid and efficient immune response. After oral administration of OBL, bacterial antigens are sampled by microfold (M) cells and then by DCs resident in the Peyer’s patches of the gut-associated lymphoid tissue (Fig. 2) [14, 60, 61]. The immune cascade generated by the activated DCs initiates homing of gut immune cells to the mucosa-associated lymphoid tissue in the airways.

## Mechanisms of action of OBL: the experimental studies

**Fig. 3:**
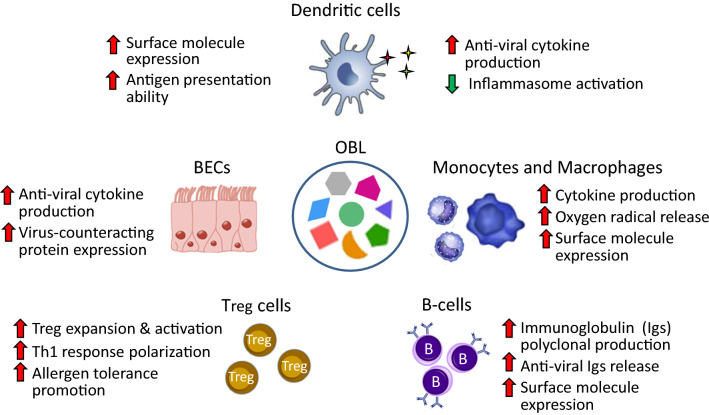
Mode of action of OBL. Modulation of target cells structure and function

Three OBL, administered by oral route, have shown to be effective in the prevention of viral infections in children: LW50020, RU 41740 and OM-85. The product that has been more extensively evaluated in recent, good quality studies is OM-85 [14, 60]. Studies performed in vitro, in experimental animals and in animal models relevant to human diseases, demonstrate that OBL can modulate the activity of a variety of cells, including DCs, monocyte-macrophages, B-cells, Treg cells and airway epithelial cells (Fig. 3) [14, 60, 61]. Only the most relevant features that may support the clinical use of these products in the prevention of viral RTIs and wheezing–asthma inception and recurrence in childhood will be described in detail.

### OBL and modulation of DC functions

DCs are major players in both innate and adaptive responses against viruses. Studies performed in vitro and ex vivo in experimental animals demonstrate that OBL can induce DC maturation, increasing the production of anti-viral cytokines/chemokines and the expression of surface molecules involved in antigen presentation [14, 60]. In murine bone-marrow-derived DCs (BMDC) and human peripheral blood-derived DC (PBDC) cultures, through interaction with toll-like receptors and activation of NF-kB and MAPK dependent pathways, OBL can trigger the production of type I IFN-β, IL-8, CCL2, CCL20 and IFN-α, molecules involved in early immune reaction against viral infections [61, 62]. OBL-induced stimulation of murine peripheral blood derived DCs (PBDCs) increases the release of IL-12, a potent inducer of Th1 type lymphocytes, and down-regulated the “anti-inflammatory” cytokine IL-10 production [63]. To achieve optimum activation of T-cells, DCs must provide two specific signals: the first through binding of MHC II molecules, expressed on DC surface, to the T-cell receptors, and the second, through binding of DC co-stimulatory molecules CD40 and CD80/CD86 to CD40 ligand and CD28 receptors on CD4+ and CD8+ T cells [34, 64]. Exposure to OBL of mice PBDCs and spleen derived DCs led to a significant increase in MHC II, CD40 and CD86 expression [63, 65]. Interestingly, a parallel reduction was detected of inducible T-cell co-stimulator ligand (ICOSL) expression, a molecule that enhances IgE antibody class switching and Th2 cytokine release [65]. Consistently, in splenocyte cultures from mice sensitized to ovalbumin (OVA), OBL increased production of anti-viral cytokine IFN-γ but decreased IgE and IL-4 release [66]. Moreover, in an infant rat model of OVA-induced sensitization, an increased ability to develop a Th1-dependent delayed response to OVA was detected, characterized by upregulation of IgG2 and IFN-γ release and an opposite effect on IL-4 release [67]. Thus, OBL may reinforce the postnatal maturation of Th1 function, a process thought to be physiologically driven also in humans by the gastrointestinal commensal microflora [50, 51].

### OBL and modulation of mononuclear cell, macrophage, B- and Treg cell functions

In human and murine blood monocyte cultures and in murine bone marrow-derived macrophage cultures, OBL increased cytokine (IL-1, IL-2, IL-13 and TNF-α) release, adhesion molecule (LFA-1, MAC-1 and ICAM-1) expression, oxygen radicals (superoxide, nitric oxide, and nitrogen dioxide) production and killing of TNF-sensitive targets [66, 68–71]. Moreover, alveolar macrophages (AM) isolated from OBL-treated rats secreted significantly more nitric oxide and TNF-α upon in vitro stimulation with lipopolysaccharide [72]. These effects were due to a priming effect of OBL on the cells, because in vitro incubation of AM with OBL or intra-tracheal instillation of OBL did not result in altered cell activity [72]. OBL can induce an antigen-specific but also a polyclonal B-cell activation. In mice, treatment with OBL increased the levels of bacterial extract-specific IgA in Peyer’s patches and mesenteric lymph nodes [66], but also induced a polyclonal production of IgA and IgG in serum and bronchoalveolar lavage (BAL) [65]. OBL also enhance the expression of the major MHC II and the co-stimulatory CD40 and CD86 molecules on B-cell surface [65]. Remarkably, OBL induced polyclonal activation of memory B-cells with production of influenza virus- and RSV-specific IgA and IgG in serum and BAL of animals not previously exposed to these viruses. These virus-specific IgA and IgG were functionally active, and significantly inhibited viral replication in vitro [65]. T- and B-lymphocyte migration from the gut to the lung, and antigen-specific immunoglobulin production in the respiratory tract were also detected in mice after oral administration of OBL [73]. In viral infections, Treg cells play a critical role in regulating effector T-lymphocytes, NK cells, neutrophils, monocytes, and macrophages. In atopic disorders, Treg cells promote allergen tolerance, decreasing Th2 inflammatory reactions [74–77]. In splenocyte cultures from OVA-induced asthmatic mice, the OBL OM-85 induced a significant increase in Treg cell frequency. In splenocyte supernatants opposite effects were detected in cytokine concentrations: IL-4 levels were decreased whilst the levels of IL-10 and TGF-β1, molecules involved in the modulation of allergic reactions and of anti-viral inflammatory responses, were increased [78].

### OBL and human airway epithelial cell activation

In addition to providing a physical and functional barrier, bronchial epithelial cells (BEC) are actively involved in the initiation of host reactions to viral infection, through the expression of various receptors and effector proteins on their surface and the release of anti-inflammatory agents [79, 80]. Exposure to RU 41740 of human BEC line cultures, led to enhanced production of granulocyte–macrophage colony-stimulating factor (GM-CSF) and of IL-8, cytokines involved in antiviral responses [81]. In primary human BEC cultures from healthy individuals and asthmatic patients, OM-85 upregulated the expression of the virus-counteracting proteins C1q-R and β-defensin [80]. Moreover, OM-85 significantly reduced HRV-induced virus replication as with it BEC death, at least partially reduced ICAM-1 expression, the main adhesion site for HRV type A and B and stimulated β-defensin release [80]. It therefore appears that OM-85 enhances BECs capacity to directly counter viral infections, by engagement of a variety of immuno-effector cells. The possibility that this effect could be translated in vivo, mediated by the gut-lung axis, is an attractive hypothesis.

## Animal models relevant to human diseases

**Fig. 4:**
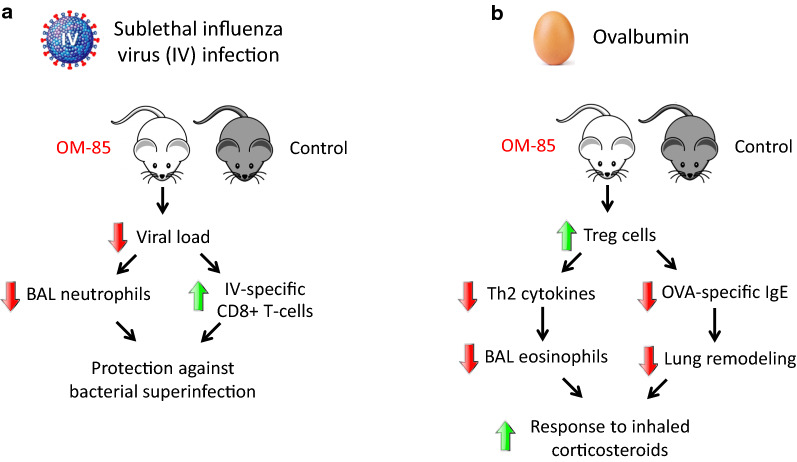
Mouse models relevant to human diseases. **a** Sublethal influenza virus infection: pretreatment with OM-85 protected mice against viral infection but also from bacterial superinfections suggesting that the anti-influenza-specific activation of the immune system was associated with an enhancement of specific anti-bacterial responses. **b** Ova-induced allergic asthma: the expansion of Treg cells induced by OM-85 was associated with a significant decrease of the Th2 inflammatory response to the allergen and enhanced the response to inhaled corticosteroids

The possible “anti-viral” activities of OBL, shown by the in vitro studies, were confirmed in a mouse model of influenza virus infection (Fig. 4a). In animals treated for 10 days and then infected with sub-lethal doses of the virus, OM-85 decreased viral load in lung tissue [65]. Analysis of airway cellular infiltrates showed a reduction of neutrophilic inflammation with an increase in CD8+ cytotoxic T-cell proportions in OM-85 treated mice [65]. In the same experimental model, on day 7 following influenza infection, mice were exposed to a sublethal dose of either *Klebsiella pneumoniae* or *Streptococcus pneumoniae* [65]. Pre-treatment with OM-85 protected mice from bacteremia and significantly reduced all morbidity signs, suggesting that the anti-influenza-specific activation of the immune responses was also associated with an enhancement of specific anti-bacterial response against pathogens, whose PAMPs are included in OBL [82]. In addition to the effect on infection, a possible beneficial effect on allergen tolerance promoted by OBL-activated Treg cells was demonstrated in an asthmatic mouse model established with OVA challenge [78]. As compared to controls, in OBL pre-treated animals, a significant decrease of serum OVA-specific IgE concentration, BAL inflammatory cells (including eosinophils) percentages and BAL IL-4, IL-5 and TGF-β1 levels was noted [78]. In contrast, a significant increase in BAL levels of IFN-γ and IL-10 was detected [78]. Their lungs showed attenuation of mucous metaplasia and eosinophilic infiltration and enhanced presence of Treg cells [78]. In a similar OVA-induced asthmatic mouse model, in addition to reducing lung inflammatory cell infiltration, an OBL (OM-85) enhanced the anti-inflammatory activity of an inhaled corticosteroid [83]. In a different asthmatic mouse model (animals sensitized with Leishmania major LACK antigen), oral treatment with OM-85 suppressed airway inflammation through IL-10- and MyD88-dependent mechanisms and induced the activation of Treg cells [85]. Furthermore, CD4+ T-cells purified from the trachea of OM-85-treated mice conferred protection against airway inflammation when adoptively transferred into sensitized mice [85]. In summary, an interesting feature of OBL that emerges from the experimental studies and contributes to understanding their efficacy and at the same time their good safety profile, is their ability to act as “immunoregulators”, rather than only as “immunostimulators”. The immunoregulatory functions of these compounds include downregulation of expression of surface molecules associated with allergic Th2 type responses on DCs [65] and of HRV infectivity on BECs [80, 81] as well as promotion of Treg cells expansion [78, 83, 84].

## Clinical studies

Several reviews and meta-analyses have shown that OBL can be effective in prevention of recurrent RTI of the upper and lower airways in pediatric populations [14, 85–89]. Reduction of incidence, severity and duration of infections, as well as of antibiotic and drug use have been reported in randomized clinical trials on preschool and school-aged children and in adolescents treated with LW50020, RU 41740 and OM-85 [90–97]. Treatments were shown to be unaffected by co-administration of antibiotics and well tolerated, with a safety profile stable in nature and frequency over long-term use [90–97]. In addition, the efficacy of OM-85 treatment was shown to be unaffected by co-administration of influenza vaccination. A study on children aged 36–59 months with recurrent RTI showed that this OBL had no effects on the immunogenicity, safety and tolerability of inactivated influenza vaccine, and conferred additional benefit in terms of absenteeism and prevalence of infections [93]. In another study the same OBL has been shown to be effective in significantly reducing infectious wheezing episodes by 38% and their duration (2 days), in preschool children [94]. In a similar study, through the 12-month study period, RU 41740 significantly reduced the mean incidence of wheezing attacks by 37.9% and the mean incidence of acute RTIs by 31.4% [91]. In asthmatic children on long-term control medications (inhaled corticosteroids), OM-85 treatment was associated with a reduction of RTI frequency, significant at the 6 and 12-month control visits. The study also showed a significant increase of serum levels of IgA, IgG and human β defensin-1, a molecule that promotes DC and T-cell recruitment [95]. When given to school-aged asthmatic children and adolescents on conventional asthma therapy over a period of 12-month, OM-85 treatment reduced the frequency of RTI, asthma attacks, and antibiotic use [96]. These clinical effects were associated with an increase in the proportion of blood NK cells and IL-10 and IFN-γ serum levels, with a rise in the IFN-γ/IL-4 ratio [96]. The latter two studies suggest that OBL prophylaxis, when combined with conventional asthma treatment, can prevent recurrent RTI and wheezing/asthma attacks, putatively by modulating the immune response against viral infections. According to a recent meta-analysis and other combined study analyses, the greatest clinical benefit of OBL in wheezing and asthma should be expected in young children, in whom a history of frequent RTIs is more common, suggesting that infants could be an ideal population to test [60, 86–89, 96]. Nevertheless, based on the mode of action and the preliminary evidence on OBL in wheezing illness and asthma, some researchers have started testing OBL even in primary prevention in at risk infants/children. In a recent randomized clinical trial, at-risk infants (positive parental history of asthma and allergies) were treated with OM-85 during their first two winter-viral-seasons, and followed for a 3rd year, off treatment [98]. The frequency of severe lower RTI was significant lower in the 1st winter of the child’s life in those receiving OM-85. Moreover, the time to the 1st sLRTI was significantly longer, and the cumulative frequency of sLRTIs and the number of days with sLRTI symptoms were significantly lower for infants in the active group, suggesting a reduction in the overall inflammatory burden in the lower airways during this crucial period of early lung growth [98]. Finally, the ORBEX study, a large, multicenter, National Institute of Health-funded, randomized controlled trial, is currently ongoing in the USA. The study encompasses young (6–18-month-old) children at high asthma risk, i.e. having atopic dermatitis and/or parents or first-degree siblings with asthma [99]. The primary objective of this study is to evaluate whether an OBL, given for 10 days monthly for two consecutive years, can increase the time to occurrence of the first episode of wheezing lower respiratory tract illness during a third observation year, off therapy. The preliminary results of this trial are expected by December 2023.

## Conclusions

OBLs appear to act on both the innate and the adaptive arms of the immune response, conferring efficacious broad immunoglobulin-related and cell-mediated immunity to the respiratory system. Additionally, experiments performed on BECs show that OBLs can also directly reduce viral infections, regardless of the engagement of the inflammatory and immuno-effector cells. Mechanistic data on the mode of action and results of clinical studies in pediatric patients can sustain the rationale for use of OBLs in the prophylaxis of viral RTIs and associated wheezing/asthma exacerbations. In addition to improving the quality of life of young patients and their families and to limiting the economic and social impact of these frequent respiratory disorders, the reduction of the frequency and severity of RTI is expected to decrease inappropriate use of antibiotics. Reduced prescription of antimicrobial drugs will have positive effects on the preservation of the physiologic gut microbiota composition and diversity, and importantly on reducing development of drug resistance. Ongoing (e.g. ORBEX) and future studies will show whether early OBL treatment can also be effective in prevention of long-term respiratory consequences of early and severe or recurrent RTI in childhood and will contribute to clarify the effect of OBLs both on immune system effectors and on microbiota and its metabolites.

## Data Availability

Not applicable.

## Abbreviations

AM: Alveolar macrophages
BAL: Bronchoalveolar lavage
BEC: Bronchial epithelial cells
BMDC: Bone-marrow-derived dendritic cells
CDHR3: Cadherin-related family member 3
CCL2: Chemokine (C–C motif) ligand 2
CCL20: Chemokine (C–C motif) ligand 20
DC: Dendritic cells
GM-CSF: Granulocyte-macrophage colony-stimulating factor
HRV: Human rhinovirus
ICAM-1: Intercellular adhesion molecule-1
ICOSL: Inducible T-cell co-stimulator ligand
IFN-γ: Interferon gamma
LFA-1: Lymphocyte function-associated antigen 1
MAPK: Mitogen-activated protein kinase
M cells: Microfold cells
MAC-1: Macrophage antigen-1
MHC II: Major histocompatibility complex class II molecules
NK cells: Natural killer cells
OBL: Oral bacterial lysates
OVA: Ovalbumin
PAMPs: Pathogen-associated molecular patterns
PBDC: Human peripheral blood-derived DC
PRRs: Pattern recognition receptors
RTI: Respiratory tract infections
RSV: Respiratory syncytial virus
TGF-β: Transforming growth factor-β
Th17 cells: Interleukin IL-17-producing T helper cells
TNF-α: Tumor necrosis factor-α
Treg cells: Regulatory T-cells
NF-kB: Nuclear factor kappa-light-chain-enhancer of activated B cells
